# Perceptions, experiences, and motivation of COVID-19 vaccine trial participants in South Africa: a qualitative study

**DOI:** 10.1186/s41073-024-00148-6

**Published:** 2024-07-29

**Authors:** Thandeka Nkosi, Chanelle Mulopo, Bey-Marrié Schmidt

**Affiliations:** 1 School of Public Health, University of the Western Cape, Bellville, Cape Town, South Africa; 2 Desmond Tutu HIV Centre, University of Cape Town, Cape Town, South Africa; 3 Health Systems Research Unit, South African Medical Research Council, Cape Town, South Africa

**Keywords:** Informed consent, COVID-19, Clinical trials, Study participation

## Abstract

**Background:**

The informed consent process is an important step in conducting ethical clinical trials, as it ensures that research participants are aware of their rights and responsibilities in clinical trials. This study explored participants’ perceptions, experiences and the factors motivating their participation in a COVID-19 vaccine trial in South Africa.

**Methods:**

This descriptive qualitative study was conducted among twenty-five adult participants (18 to 64 years old) who participated in a COVID-19 vaccine trial in South Africa. Three focus group discussions and fifteen semi-structured interviews were carried out. Data were collected at a Clinical Research Site located in Prince Mshiyeni Memorial Hospital, in Umlazi Township, Durban, South Africa, where the COVID-19 vaccine trial participants were initially enrolled. Data were analysed iteratively using a thematic analysis approach.

**Results:**

Four key findings emerged: 1) Participants who experienced an event (such as tested positive for COVID-19) during the clinical trial were more likely to talk about the informed consent more thoroughly compared to the other participants. 2) Participants understood the purpose of informed consent process better when it was repeated multiple times throughout the course of the trial. 3) Where participants did not recall or understand various elements of the informed consent, participants were likely to create their own interpretations. 4) Factors influencing participations in trials were reimbursement for participation, access to health care, protection of family members, and ability to socialize without fear of COVID-19.

**Conclusion:**

Overall, the findings show that the informed consent process should be regarded as an ongoing process rather than a once-off event that only happens at the start of a clinical trial. An understanding of participants’ perspectives, experiences, and motivations for participating in clinical trials, can help trial staff strengthen the consent documents and processes.

**Supplementary Information:**

The online version contains supplementary material available at 10.1186/s41073-024-00148-6.

## Background

There has been an increase in the number of clinical trials being conducted in low and low-middle income countries (LMICs) [1]. The low cost of conducting a clinical trial in LMICs, the availability of eligible participants, and LMICs’ improving regulatory and operational capacity are some of the factors that have led to the increasing number of clinical trials being conducted in LMICs [1, 2].

Despite the various factors that have contributed to the increasing number of clinical trials being conducted in LMICs, there are ethical concerns around participation [3]. Some of the ethical concerns are related to the characteristics of those who are targeted to participate in trials, that is, their lack of or low level of education, their economic status (e.g. poverty), and lack of access to quality health care [4].

Informed consent is a key principle of ethical research, and its goal is to ensure that potential participants are fully informed of their roles and responsibilities before agreeing to participate in a research study [5, 6]. To ensure that the participants are informed about the potential risks and benefits of the intervention being tested, the informed consent should contain simple language and detailed information that can help the participants make an informed decision [7]. Legibility, formal quality, and compliance are crucial for enhancing transparency and trust between researchers and participants, upholding ethical standards in clinical trials.

A 2020 study on non-surgical procedure consent forms identified poor readability and formal quality, emphasizing the crucial role of nurse involvement in improving patient decision-making [8].

In 2023, another study found that informed consent forms in critical care and surgical areas of a Spanish county hospital exhibited deficient formal quality and inadequate compliance, particularly concerning personalized risks and complete identification [9].

Both studies highlighted deficiencies in providing personalized risk information and complete identification, advocating for enhancements to improve patient autonomy and information (8; 9). Recommendations included increased nurse involvement to enhance the compliance and quality of informed consent forms (8; 9).

In South Africa, the ethical principle of informed consent is captured in both the Constitution and the National Health Act 2003 [10, 11]. They establish the language and literacy standards required for conveying information [11]. Language barriers are common in a diverse country like South Africa that has 11 official languages.

Even though guidelines for informed consent are already available, ensuring compliance can be challenging. The need for improving the informed consent process has been documented by various studies [12–17].

A study conducted to gather COVID-19 patients’ perspectives on research participation identified factors that influenced them to participate in COVID-19 related clinical trials [18]. Factors such as the feasibility of the trial, autonomy, and privacy concerns affected the willingness of patients to participate [18]. In the context of COVID-19, they also preferred the use of virtual and remote methods for the recruitment and consent process [18]. The use of electronic informed consent was suggested as a potential alternative to the paper-based method. This digital procedure was used during the course of the COVID-19 pandemic to ensure that the clinical studies continued. [19, 20]. One example of an alternative consenting method is teleconsenting [20]. Teleconsent, through virtual methodology, allows for the informed consent process to occur entirely remotely for participants [20].

The objective of this qualitative study was to explore the experiences, perceptions, and factors that motivate individuals to participate in COVID-19 clinical trials in South Africa.

## Methods

### Study design

This was a qualitative study based on a grounded theory approach which consisted of focus groups and semi-structured interviews with participants who took part in a COVID-19 vaccine trial in South Africa. Data were collected between December 2021 and January 2022. Ethics approval was obtained from the Biomedical Research Ethics Committee of the University of the Western Cape (Reference Number: BM21/10/22).

The COVID-19 vaccine trial included two cohorts: HIV-negative and HIV-positive participants. This was a Phase 2a/b, randomized, observer-blinded, placebo-controlled study designed to evaluate the efficacy, safety, and immunogenicity of the SARS-CoV-2 rS vaccine with Matrix-M1 adjuvant. Cohort 1 consisted of healthy HIV-negative adult subjects, while Cohort 2 included medically stable HIV-positive adult subjects. The trial aimed to determine if adults living with and without HIV in South Africa could be protected from COVID-19 by this new vaccine. Blood samples for HIV testing were collected at the initial screening for inclusion in the study, with a repeat HIV test administered at the 6th month following the initial vaccination. This testing ensured proper cohort classification and monitored the safety and immune responses of the participants. Each visit included a pregnancy test, blood drawing, and a swab for COVID-19 testing. By taking part in this trial, participants were reimbursed for their time, including blood tests, procedures, and travel expenses. The minimum amount paid was R300 per visit in cash.

### Study settings

The COVID-19 vaccine trial was carried out at four different sites in South Africa. One of the COVID-19 vaccine trial sites, was Prince Mshiyeni Memorial Hospital, situated in Umlazi Township, where this research study was conducted.

Our study was carefully designed with consideration of various factors to optimize research outcomes while balancing logistical constraints and resource availability. Although we acknowledge the potential benefits of including participants from multiple sites in enhancing the transferability of findings, we believe that a single-site approach offered several distinct advantages for our study. Limiting the study to a single site allowed for a more controlled research environment. With a single-site approach, we ensured consistent implementation of study protocols and procedures, thereby increasing the reliability of our results. Additionally focusing on a single site enabled a deeper exploration of the specific context, dynamics, and nuances present, providing valuable insights into the phenomena under investigation.

Umlazi Township is in the south-eastern part of KwaZulu-Natal province, South Africa. It has a population density of around 8530 people per square kilometre. It is one of the country's largest urban areas and is bordered by the Mlazi River and the city of Durban [21]. Umlazi is a limited resourced community, with over 100,000 registered households. In addition, 49, 9% of the households have piped water, and 90% of the residents use electricity for their homes. About 40% of the population is below the age of 18, and 3, 4% are not in school. Umlazi is 99, 4% black and 91, 4% of the population are isiZulu speaking, 3% isiXhosa and 2, 1% English speaking [22].

Prince Mshiyeni Memorial hospital provides health care services to the Umlazi community. With a capacity of over 1075 beds, it can serve the area up to and including parts of the Eastern Cape [23].

### Sampling

Our study population included adults (18–64 years) who had participated in the COVID-19 vaccine trial conducted in Umlazi, Prince Mshiyeni Memorial Hospital. We sampled twenty-five participants. Fifteen participants were sampled for individual interviews and ten participants were sampled for focus group discussions (two focus group discussions with five participants each). None of the participants who took part in the semi-structured interviews were included in the focus groups. To confirm data saturation, a third focus group with six participants was conducted, including two participants from the semi-structured interviews and two from each of the initial focus group discussions.

In this study, purposive sampling was employed, using maximum variation to ensure diversity across participant demographics, including age, gender, and socioeconomic status. We used a list of participants who had exited the COVID-19 vaccine study to select participants for this study. From that list, participants were selected systematically for data collection until the required sample size was reached and data saturation was achieved. This approach allowed for the exploration of diverse perspectives and experiences within the sample population. These methods were chosen to capture a wide range of viewpoints and experiences relevant to the research question (File 1). By employing these purposive sampling techniques, the study aimed to enhance the richness and depth of qualitative data collected, thereby strengthening the validity and credibility of the findings.

### Data collection

The COVID-19 vaccine trial’s information sheet and informed consent form were used to develop questions for this study’s data collection guides. For example, it included questions related to participants’ perceptions of the elements described in the consent form; their perceptions of the comprehensiveness of the information provided in the consent form and the factors motivating participation. This study’s interview guide was developed in English and translated into isiZulu, as these are the two commonly spoken languages in Umlazi Township. Informed consent for this study was obtained from participants in their language of choice, either English or isiZulu.

### Data analysis

Audio recordings of the 15 semi-structured interviews and 3 focus group discussions were saved on a password protected computer and USB. Recordings of the interviews and focus group discussions were transcribed verbatim and translated to English. Thematic analysis was used to analyse the data [24]. The first author (TN) read through the transcripts (with the recordings) to ensure that the translations were accurate and to re-familiarise herself with what participants said. Thereafter, transcripts were coded using NVivo 12 (RRID: SCR_014802) [25]. Following this, the codes were grouped into categories to find patterns. The categories that had a common pattern were then grouped together to generate themes and sub-themes. The themes and sub-themes were reviewed and revised according to feedback from the other two authors (CM and BMS). The final themes and sub-themes were written up as findings addressing the study’s objectives.

### Researcher characteristics, reflexivity, and involvement in the study

The first author (TN) received training in interviewing in her role as a study coordinator of the COVID-19 vaccine trail. She developed the interview guide with guidance from the other two authors (CN and BMS). During the interviews, the first author was able to rephrase questions and use additional probes to ensure that participants understood the questions and provided rich information. It was also beneficial that she had built rapport with the participants during the COVID-19 vaccine trial.

It is important to note that the first author's participation in the main trial primarily involved administrative and logistical support and did not extend to decision-making processes or data analysis in the COVID-19 vaccine study. However, we recognize that the mere association may still raise questions about objectivity. To mitigate any potential bias, rigorous measures were implemented throughout the study to uphold the integrity and impartiality of the research process. This included involving BMS and CM in data analysis, maintaining transparency in reporting methodologies, and engaging in regular discussions to critically evaluate findings and interpretations. Additionally, efforts were made to separate the roles of the first author in the main trial and this study to minimize any influence on data collection and analysis.

### Rigour

To ensure that the research findings of this study accurately depict the nature of the phenomenon, the researcher applied the four criteria set forth by Lincoln and Guba (1985): credibility, transferability, dependability, and confirmability. Credibility, which concerns the accurate representation of participants’ voices and experiences, was achieved through peer debriefing [26]. Peer debriefing is an integral part of the research process, involving discussions of data collection and analysis with the supervisor to obtain feedback on research processes [26]. Additionally, triangulation was conducted by cross-checking data from individual interviews with focus group discussions. This process included a preliminary analysis after the interviews to verify and confirm issues during the focus group discussions, which led to the addition of another focus group discussion.

Transferability was enhanced by employing a purposive sampling method and providing detailed descriptions of participants, settings, contexts, and their responses to interview or focus group questions. Dependability was achieved by having a peer qualitative researcher and the supervisor review the anonymized transcriptions to validate the identified themes and descriptors. The researcher maintained a notebook to record all decisions made throughout the research process and a reflexive journal to document decisions and thoughts, minimizing bias and ensuring confirmability. Quotes from participants were used where appropriate in the study write-up [26].

### Ethical considerations

COVID-19 safety protocols were strictly adhered to during interviews, conducted in a designated area with privacy screens. Participants were informed about the study during their vaccine trial exit visit and provided explicit, signed consent. Information sheets and consent forms were available in English and isiZulu. Participants were briefed on study objectives, risks, benefits, privacy, confidentiality, and their right to withdraw at any time. Focus group confidentiality agreements were implemented. Measures were taken to ensure participant confidentiality and data security, including password-protected storage and adherence to the South African Protection of Personal Information Act (POPIA). All data will be securely stored for five years and disposed of in compliance with regulations. The researcher will continue to uphold participants' rights under POPIA, including access, correction, and deletion of personal information.

## Results

The study's results highlight several key themes regarding participants' perceptions, experiences, and motivations in participating in a COVID-19 vaccine trial. Firstly, participants demonstrated varying levels of understanding and recall regarding the informed consent process, particularly concerning changes made throughout the trial. While some could recall specific details, others struggled to remember or comprehend the process fully.

Secondly, participants generally grasped the key ethical issues of the trial, including the investigational nature of the vaccine, the placebo concept, and the purpose of storing leftover blood samples for future research. However, there were misconceptions about the reasons for storing samples, with some participants believing they would be donated to hospitals for transfusions.

Thirdly, participants discussed the influence of their communities and families on their trial participation, with some facing scepticism and misinformation, particularly regarding conspiracy theories about the vaccine.

Lastly, factors affecting participation included financial reimbursement, access to healthcare services, and concerns about COVID-19 infection and its potential side effects. Despite initial motivations often being financial, participants recognized additional benefits such as free healthcare services, routine testing for HIV and COVID-19, and access to family planning resources, which contributed to their continued participation. Overall, the results underscore the multifaceted considerations influencing individuals' decisions to participate in COVID-19 vaccine trials and highlight the importance of addressing misconceptions and providing comprehensive support to trial participants.

Socio-demographic characteristics of participants are shown in Table 1.

**Table 1:** Socio-demographic characteristics of participants

Variables	Categories	Number (n)
Gender	Female	17
	Male	8
Age	18–24	6
	24–34	11
	35–44	4
	45–54	2
	55–64	2
Level of education	Still in Secondary school	1
	Did not complete primary school	7
	Completed Secondary School	12
	Some tertiaries but not completed	3
	Undergraduate degree	2
	Post graduate degree	0
Language	isiZulu	13
	English	12
Employment status	Employed Full time	0
	Employed Part time	5
	Unemployed	14
	Secondary School student	1
	Tertiary Student	4
	Other	1

Thematic analysis of participant interviews and focus group discussions revealed several key themes elucidating participants' perceptions, experiences, and motivations in participating in the COVID-19 vaccine trial. Through the analysis, four overarching themes emerged, shedding light on the multifaceted considerations influencing individuals' decisions to participate in the trial: (i) Participants’ perceptions of the informed consent process; (ii) (Mis)Understanding of the elements of informed consent; (iii) Involvement of community and family members in a trial; and (iv) Factors affecting participation in the COVID-19 vaccine trial study. Each of these themes has been developed by triangulating data from the semi-structured interviews and focus group discussions, and relevant quotes have been included to support each theme.

### Theme 1: Participants’ perceptions of the informed consent process

In the trial, participants were reconsented three times when: 1) the age and trial sample size were changed; 2) a repeat HIV test was included at the 6th month following the initial vaccination; and 3) new safety information on the trial vaccine was distributed to participants via a memo. The memo was discussed with participants telephonically and, if they came in for their next scheduled visit, it was discussed in person.

There was poor recall of the changes that occurred during the trial. Of the 25 participants interviewed, only one participant was able to recall all three changes to the informed consent documents.

*“If I remember correctly the first few months, we use to do visits every week, but it changed as time goes and then I think age, more old people allowed…” (Male, 21 years old)*

While most participants recalled signing another information leaflet and informed consent form, they did not remember why.

*“That time we were changing the vaccine because the first time we vaccinated we did sign the forms, but I don’t remember what the first vaccine was called but the second vaccine we got was called Novavax” (Female, 29 years old)*

Other participants did not recall the content but remember being reconsented by the first author (TN).

*“I don’t remember well but there was a second one we signed. I think either last year or this year. I honestly don’t remember what we were signing for” (Female, 25 years old)*

Participants were more likely to recall recent events and changes in the trial. For example, they could recall receiving updated safety information, which was the third (last) change to the informed consent process.

### Theme 2: (Mis) Understanding of the elements of the informed consent process

Participants were asked questions about the key ethical issues of clinical research; outlined in the International Council for Harmonisation (ICH)—GCP E6 (R2) and South African guidelines for Good Clinical Practice (GCP). The questions covered various aspects related to the trial and their involvement, such as the investigational nature of the trial, their rights and responsibilities in the trial, the ways in which confidentiality was upheld, and their compensation for participating in the trial. Most participants were generally able to understand the various elements of the trial; for example, they understood the purpose of the trial and why it was conducted.

*“The Novavax study was the research program for COVID-19 vaccine, since there are other vaccines like Johnson & Johnson and many others, so this study was to check if Novavax vaccine can work” (Male, 36 years old)*

Participants in both the semi-structured interviews and the focus group discussions remembered the *placebo*. They understood that there were two injections; either a vaccine (the investigational product) or a placebo of salt water (sterile normal saline). Almost all participants were able to explain that the sterile normal saline was a placebo and almost all participants understood that a placebo was a “fake vaccine”.

*“It’s a program about COVID-19 vaccine, whereby two vaccines were shown to us which are placebo and Novavax vaccine. Placebo is made up of fake water and salt.” (Male, 23 years old)*

As part of the main trial, permission was sought to store any leftover blood for future vaccine research by way of an information leaflet and informed consent form. This consent for storage was obtained during the initial consent process at the beginning of the study. The decision to store leftover blood for future use did not affect participation in the trial or the care that the participant received at the research site. Blood draws were required for both HIV testing and vaccine-related assessments. Participants were not tested for any other conditions beyond those specified for HIV and the COVID-19 vaccine trial. Participants remembered being informed of and consenting to the storage of leftover samples. They understood that their samples would be stored and used for future research; however, there were misconceptions about the reasons for storing leftover samples and using them in future research.

*“They are sent to hospital for people who need blood, to assist them…” (Female, 33 years old)*

*“They were stored to help others maybe who had a shortage of blood. But only those that matched my blood type that is healthy and does not have any illnesses” (Female, 33 years old)*

Participants were asked to provide examples of how samples would be used in the future. Many participants believed that samples would be donated to patients in hospitals who require blood.

Participants understood the purpose of informed consent better when elements of the informed consent process were repeated to them throughout the trial, such as the key procedures of the trial and the reason why they were being performed. Participants who experienced an ‘event”, that is, those who tested positive for COVID-19 during their participation in the clinical trial, talked about the informed consent process more thoroughly. These participants were most likely to mention the Flu Pro, which is a questionnaire, provided to them for recording the severity of COVID-19 related symptoms over 10 days.

*“If you were not feeling well, you could, if you sick and maybe have the flu, you can call them and let them know that you are unwell and then they would fetch you and bring you to the clinic and give you medication. You return for them to see if you are getting better and if the medication is working. You also were expected to complete a form to track your symptoms each day in the 10 days” (Female, 53 years old)*

Those participants that did not experience a COVID-19 related event, either did not remember what process was followed when an event occurred (including testing for COVID-19), or they provided responses that were more generic about COVID-19 or even information that was not specific to the trial.

*“They would take me to hospital, and I would get treated until I was better.” (Female, 29 years old)*

### Theme 3: Involvement of community and family members in a trial

Participants spoke about their experiences during the trial and how their community and family members responded to their participation in the trial. Several dynamics emerged, namely, those related to conspiracy theories and misinformation about the COVID-19 vaccine, trial procedures, and continued trial participation. In certain cases, participants reported that their families did not support their participation in the trial because they did not understand it.

*“They couldn’t understand at home and wouldn’t hear of it. But I told them that this was research… to prevent covid. Any other person who would ask what I am doing at Mshiyeni, I would tell them that it’s a covid thing to help us not get covid…” (Female, 25 years old)*

Participants were also used by their families to test ‘theories’ that were circulating within their communities, such as the coin test. There were videos on social media platforms, such as Instagram and TikTok, where individuals who claimed to have been vaccinated would use money, such as the R2 coin, and stick the coin where they had received the vaccine. The claim was that the COVID-19 vaccine contained something magnetic and was proof of the presence of a microchip used by the government to track people. One participant had family members stick a coin on their vaccinated arm, which confirmed that the coin did not stick. Although the coin did not stick to their vaccinated arm, the participant still sought confirmation from the interviewer as to whether or not the coin theory was true.

*“They say that after vaccinating, if you put money on the vaccinated arm it will stick. …yes, at home they tested it with me, and the money did not stick* (*Male, 31 years old)*

Participants said that their community had no trust in the COVID-19 vaccine. There was a belief that participants would not be receiving a COVID-19 vaccine but that they were being injected with the virus itself or ‘something’ that could cause harm to them and result in death.

*“We did not know how everything would turn out, especially starting something and people saying that this is not the vaccine this is something that could cause us to maybe demise (Female, 26 years old)*

Although responses from family members was mostly negative, participants shared some positive experiences. Participants felt that after being part of the trial, the onus was on them to go back to their families and community and show them that they were doing well and dismiss the rumours. Participants noted the importance of using what was in the informed consent and what was discussed with them by the trial team to give feedback to their community.

*“We shouldn’t go back now and frighten others. Like let me go back and explain the study like it was explained to me and not add extras things that were not mentioned… Let’s talk what’s in the study and direct and not make up things. I think that it’s important that we as participants do that and where we cannot answer refer people” (Male, 58 years old)*

Participants had a different experience when it came to interacting with their friends about the trial, as their friends required little convincing or motivation about the vaccine and trial. Participants said they did not have to provide great details about the trial but were able to influence their friends because they were already part of it. One participant mentioned that she and her friends generally share opportunities to make money, and the trial was such an opportunity.

*“So, me I said there is something that I have joined. Sis Thandeka, you know that as friends we always discuss with each other that if there is anything that has connection that involves money we should always plug each other…” (Female, 25 years old)*

### Theme 4: Factors affecting participation in the COVID-19 vaccine trial

Reimbursement played an important role in participants’ willingness to participate in the trial. When participants were asked why they continued in the trial even when they had reservations about it, their response was because of the money (reimbursement).

*“In honest truth money motivated me [laughs]…” (Female, 26 years old)*

*“For me if the money was not there, I don’t think it would have been that interesting and it would not have had that pull to attract someone because firstly there is no one who just wants to be drawn bloods..” (Female, 36 years old)*

Many participants did not have jobs and sought alternative income. Participants felt that reimbursement provided the financial relief that many of them needed.

*“..because as a person who was sitting at home and not working, combined with talk of the covid 19 pandemic, and the fact that it was a free program…it might be better that I come and enter the research program, especially being unemployed” (Male, 53 years old)*

Some of the trial participants did not like the various procedures that were involved in the trial, such as blood draws and swabs. However, there were also individuals who did not complain and decided to join the trial, as they wanted to continue their social life.

*“What made me sign, I saw that this this has sense for example it checks covid a lot. See with me I love going out, covid or no covid I still would go out. So, it helped me joining this research because I knew where I stood. If I felt like something was off, I would always call and get tested. The testing for covid was what I loved.” (Female, 21 years old)*

Participants were also concerned about the loss of community members through death. They wanted to protect their relatives and themselves.

*“What made me sign is that a lot of people that I was close with passed on due to covid and I saw that I needed to protect myself and my children because I also go out a lot.” (Male, 58 years old,)*

Participants noted fear as one of the key factors that they had experienced, but this did not prevent them from participating in the COVID-19 vaccine trial. Fear was experienced in the form of a fear of the unknown and the fear of potential side effects of the vaccine. Participants already enrolled in the trial who had experienced side effects were used as a reference point for those who had not yet joined and/or received the vaccine or placebo.

*“They said they had gotten sick and there were scary things resulting from the vaccine coming out of there body. However, after I had been vaccinated, I saw that I did not have anything” (Female, 21 years old)*

One participant stated that she joined the trial due to the fear of getting COVID-19, and her reservations and fear were also caused by the fact that the vaccine was being tested in humans after only being tested in monkeys.

*“I really don’t want to lie, I was scared but then I was like you know what fine, this disease is already here, and invading let me try and see how this goes… this vaccine had never been tested on any one before only in monkeys. Although monkeys are similar to humans, they still not humans so I was scared to join” (Female, 26 years old)*

Although money was the initial motivation for many participants, they said that they found out about the many benefits of participating in the trial after going through the consent process, such as ease of access to care without having to wait in long queues like at their local clinics. These benefits facilitated continued participation in the trial.

*“I got here, and it was explained to me, I realized ok, besides the money there are other things that would benefit me that aren’t associated with the money.” (Female, 21 years old)*

The trial also provided additional benefits. A number of participants recognized the benefit of being able to access free healthcare and medications through the trial.

*“And also, another thing that helped, the fact that we were here under research, once you saw you had an issue, they would be able to take care of you here (at the clinic). Because should you feel sick, you would call, and they would come to you to check on how you were doing. I will get care that is free and right…we would get the medication, care, and expertise that showed us you cared for us.” (Female, 21 years old)*

Female participants felt that they benefited from the trial because of the routine pregnancy tests and family planning being offered as part of the trial’s procedures. This meant that participants did not need to attend local clinics.

“Secondly, like the family planning like I said it meant not going to the clinic and standing long queues… Like you will find you missed a period and now you scared and not sure if you pregnant I could get a pregnancy test here.” (Female, 26 years old).

Through the trial, the participants were able to check their HIV status as well as whether or not they had COVID-19.

“Another thing was the ability to check my status. A lot of people are afraid to check and know of which these were all things that were beneficial for me that allowed me to continue in the study.” (Female, 29 years old).

During the initial stages of the COVID-19, participants also appreciated knowing if they contracted the virus.

“The benefit of this program is that you know your status all the time and that when we first came here corona cases were high and people were panicking so for me it helped me to know that I am not infected with COVID-19.” (Male, 20 years old).

## Discussion

Our study has shown that participants who experienced an event such as testing positive for COVID-19 during the trial showed a better understanding of the informed consent process. Those who were unable to recall the various elements were more likely to create their own interpretations. Various factors motivated people’s decisions to participate in the trial, including reimbursement, protecting their families, and maintaining a social life.

This study revealed that most participants understood the purpose of the COVID-19 vaccine trial, however, there were misunderstandings when it came to the elements of informed consent. The trial had two injection periods, which seemed to be a major cause of confusion for participants. Most participants thought that they received two different types of COVID-19 vaccines during the two injection periods, while they were actually only receiving one type of vaccine. While other participants confused the placebo for the vaccine, and believed that at the second injection period, they were receiving an actual vaccine, which was not the case. Similarly, systematic reviews on studies assessing informed consent concluded that certain elements of informed consent, including randomization, experimental nature, and the availability of alternative treatments, are universally difficult for participants to understand [27, 28].

As such, future studies could focus on the effectiveness of current informed consent processes. Future studies could also investigate the effects of shortening informed consent processes, and how to clarify informed consent for sample storage and use for future research.

The heightened understanding of informed consent among participants who tested positive for COVID-19 could be attributed to several factors. Firstly, individuals who have experienced COVID-19 first hand may have a more personal and immediate understanding of the importance of medical interventions, such as vaccines, in combating the disease. This direct experience may lead to increased health literacy and engagement with information related to the trial, including the informed consent process. Secondly, individuals who have been diagnosed with COVID-19 may have had more interactions with the research study team and exposure to medical information, which could contribute to their comprehension of the informed consent documents. These interactions may have provided opportunities for additional education and clarification regarding the trial procedures. Furthermore, the experience of testing positive for COVID-19 may have heightened participants' awareness of their own health status and the potential risks and benefits associated with participating in the vaccine trial. This heightened awareness and sense of personal relevance may have motivated individuals to pay closer attention to the details of the informed consent process and seek clarification when needed.

Overall, the direct experience of COVID-19 could have served as a catalyst for participants' increased understanding of the informed consent process, emphasizing the importance of personal relevance and contextual factors in shaping comprehension and engagement with medical information.

This study found that participants wanted to protect their families from COVID-19 and contribute to a cause that could benefit their broader community. Participants felt that through their participation they could dispel misinformation and reduce fear among their family members and communities. They anticipated that their 'successful' participation in the trial would increase trust in the COVID-19 vaccine, thereby increasing future uptake of COVID-19 vaccines by their families and communities. Similarly, a study conducted in Egypt, Saudi Arabia and Jordan gathered information about the public's attitudes toward participating in COVID-19 clinical trials [29]. A questionnaire was created and distributed through social media channels [29]. The study identified and analysed the factors that influenced these attitudes towards participating in COVID-19 clinical trials and found that altruism was one of the factors that influenced people’s willingness to participate in COVID-19 clinical trials [29]. These results suggest that altruistic motivations can be used to encourage people to participate in clinical trials, even when the risks are high.

Additionally, participants in this study were motivated by access to health care through the trial. They referred to the trial as “personal medical aid”, a form of insurance that can help them access health care and avoid health care related expenses. Women mentioned that they benefited more from participating in the trial, as they could more easily access family planning services through the trial than at local clinics. In addition to the financial rewards from trials, access to health care, was another important motivator in Egypt, Saudi Arabia and Jordan [29] and Qatar [30]. Similarly, in Qatar, availability of healthcare and altruism were factors that influenced the people's perceptions and willingness to participate in clinical trials [30].

Participants' decision to enrol in the trial was influenced by multiple factors, including altruism, access to healthcare, and the desire to protect themselves and their families from the impacts of COVID-19. While financial compensation served as an incentive, it was not the sole motivator for participation. Altruism emerged as a significant driver, with participants expressing a strong sense of communal responsibility and a desire to contribute to public health efforts. Many participants viewed their involvement in the trial as an opportunity to help combat the pandemic and potentially benefit their communities. In addition to altruism, access to healthcare through the trial was cited as a compelling motivator, particularly among individuals facing barriers to traditional healthcare services. The prospect of receiving the vaccine and safeguarding their families and communities from severe illness further incentivized participation. Furthermore, participants highlighted the importance of being able to resume social activities without the fear of contracting severe COVID-19. This desire for normalcy amidst the pandemic underscored the complex interplay of motives driving participant enrollment in the trial.

## Limitations

A limitation is that the first author (TN) was involved in the recruitment and informed consent process of the COVID-19 vaccine trial, and was working as a study coordinator at the trial site when this study was conducted. Although she was involved in both the trial and this study, she ensured that participants sampled into this study knew that this is a new and different study from the COVID-19 vaccine trial. Secondly, we only recruited participated from one trial site (i.e. the COVID-19 vaccine trial) because of resource and logistical constraints. However, we selected suitable participants for addressing the research objectives, and we provided rich contextual information for transferability to other trial settings. Thirdly, given the small sample size and the specificity of the context, it is advisable to exercise caution when applying the findings of this study. Lastly, the significant gender imbalance, with more than twice as many women as men participating, may affect the generalizability and interpretation of the results.

## Conclusion

This study provides valuable insights into the factors that influenced participation in a COVID-19 vaccine trial. Our findings suggest there is a need for greater focus by researchers on how participants perceive and interpret the information they receive as part of the informed consent process in clinical trials. To make sure that participants have a clear understanding of the trial, it is important that participants are able to recall all elements of the informed consent. Informed consent therefore needs to be an ongoing process throughout the trial, and recall of information needs to be assessed at various time points across a trial. This should focus on fundamental elements of informed consent, particularly those that may cause confusion, such as which vaccine is being tested. To facilitate this, more studies exploring tools that assess participants' understanding of clinical trials and accompanying informed consent processes are needed.

### Supplementary Information

Supplementary Material 1.

## Data Availability

Due to sensitivity of the information, the data cannot be publicly shared, but has been provided to the journal editor and reviewers during the peer review process.

## Abbreviations

LMICs: Low-Middle income countries
IC: Informed Consent
ICH: International Council for Harmonisation
GCP: Good Clinical Practice
