# Supplementary material for: Perceptions, experiences, and motivation of COVID-19 vaccine trial participants in South Africa: a qualitative study
Source: Res Integr Peer Rev. 2024 Jul 29;9:8. doi: 10.1186/s41073-024-00148-6 (PMC11285467; doi:10.1186/s41073-024-00148-6)
Supplement: Supplementary file 1 — Supplementary Material 1. [file 41073_2024_148_MOESM1_ESM.docx]

| 1. Please tell me what you understand about the COVID-19 vaccine study and why it is being done? |
| --- |
| 1. What were you told would happen if you test positive for COVID-19 while in the study? |
| 1. Were there any changes to the informed consent form? Do you remember them? |
| 1. What happens to all the information that is collected, and the specimens collected during the duration of the COVID-19 vaccine study? |
| 1. Some people have said that there are those that join the COVID-19 vaccine study because of the money. What is your take on this? |

File 1:
